# High-performance 2D MoTe_2_-based photodetectors with superior Vis-NIR detection capability

**DOI:** 10.1039/d6ra00257a

**Published:** 2026-05-08

**Authors:** Xin Wang, Qingyu Zhou, Junkai Shen, Lin Lin, Hailing Tu, Guohu Zhang

**Affiliations:** a GRINM National Engineering Research Center for Key Materials of Integrated Circuits Beijing 100088 China wangxin@gritek.com tuhl@grinm.com zhangguohu@gritek.com; b GRINM Semiconductor Materials Co., Ltd Beijing 100088 China; c General Research Institute for Nonferrous Metals Beijing 100088 China; d School of Materials Science and Engineering, University of Science and Technology Beijing Beijing 100083 China

## Abstract

Photodetectors are crucial components for photoelectric conversion in intelligent sensing applications, and their performance can be significantly enhanced by using two-dimensional (2D) materials. In this study, we present a detailed investigation of 2H-MoTe_2_-based field-effect transistors (FETs) for photodetection. We demonstrate that air annealing at specific temperatures effectively improves charge transport properties by stabilizing the electrical polarity, enhancing transconductance by 230% and carrier mobility by 41%, primarily through defect healing in the MoTe_2_ lattice. The optimized 2D MoTe_2_ photodetector exhibits an impressive responsivity of 3.53 A W^−1^ and an external quantum efficiency (EQE) of 652% at 808 nm under 0.08 mW mm^−2^. Furthermore, the constructed MoTe_2_/MoSe_2_ van der Waals heterojunction exhibits excellent rectifying behavior and ultralow dark current (<10^−13^ A at *V*_gs_ = −30 V). It delivers a peak responsivity of 3.32 A W^−1^ at 671 nm, with an EQE of 614% and a photocurrent-to-dark-current ratio of 466 under 0.009 mW mm^−2^. These results highlight the potential of 2D materials, particularly MoTe_2_ and its heterojunctions, for high-performance visible-to-near-infrared(Vis-NIR) photodetection, paving the way for advanced applications in optoelectronics.

## Abstract 2

This work explores 2H-MoTe_2_-based FETs. An optimized 2D MoTe_2_ photodetector and a MoTe_2_/MoSe_2_ heterojunction both exhibit excellent photodetection performance, highlighting the promise of MoTe_2_-based devices for Vis-NIR photodetection.

## Graphical abstract

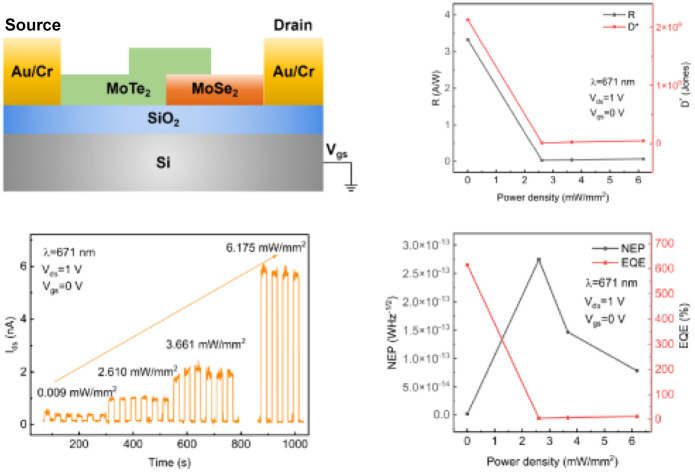

## 1. Introduction

Photodetectors, as the core components for photoelectric signal conversion, play a crucial role in information acquisition in intelligent sensing systems.^1^ With the rapid advancement of modern information technologies, there is a growing demand for photodetectors with nanosecond-level response, micron-scale dimensions, high quantum efficiency, multispectral compatibility, and cost-effective large-scale integration.^2–5^ However, traditional photodetectors are constrained by their narrowband response and complex fabrication processes, falling short of meeting these emerging requirements.^1,6^ The development of next-generation photodetectors is therefore imperative.

Two-dimensional (2D) materials, owing to their dimensional advantages, exhibit superior performance in fabrication, integration, and stability, showing broad application prospects in optoelectronic devices, spintronics, and energy catalysis.^7–12^ When material thickness is reduced to the atomic scale, significantly enhanced quantum confinement effects can impart novel physical properties.^13^ Among these, Group VI transition metal tellurides (TMTs) have attracted considerable attention due to their unique electronic structures and exotic quantum phenomena. These materials, with the general formula MTe_2_ (M = Cr, Mo, W), can crystallize in various phases such as 2H, 1 T′, and Td. This structural diversity makes them ideal platforms for exploring exotic quantum states, including the quantum spin Hall effect,^14,15^ topological superconductivity,^16^ anisotropic spin–orbit coupling,^17^ and Weyl semimetal states.^18,19^ Within this family, two-dimensional molybdenum telluride (MoTe_2_) stands out as a representative material.

As a recent addition to the monolayer semiconductor family of transition metal dichalcogenides (TMDCs),^20^ MoTe_2_ has emerged as a focal material for optoelectronic research due to its exceptional photoresponse characteristics. Experimental studies have demonstrated its superior room-temperature mobility (>100 cm^2^ V^−1^ s^−1^),^21^ addressing the performance limitations of conventional n-type 2D materials, such as MoS_2_.^22^ Monolayer MoTe_2_ is a direct-bandgap semiconductor with a bandgap of ∼1.1 eV,^23^ comparable to crystalline silicon, and has an interlayer spacing of about 0.7 nm^24^ with semimetallic characteristics.^25,26^

Significant progress has been made in MoTe_2_-based photodetectors. For instance, Huang *et al.*^27^ developed a MoTe_2_ photodetector that demonstrated broadband response across the 0.6–1.55 µm range, achieving a responsivity of 24 mA W^−1^ at 1060 nm. Luo *et al.*^28^ engineered a vertical MoTe_2_/MoSe_2_ heterojunction photodetector with exceptional performance, achieving a photocurrent-to-dark-current ratio greater than 10^4^ under white light and a responsivity of 1.5 A W^−1^. Chen's team^29^ utilized chemical vapor deposition (CVD) to fabricate MoTe_2_/MoS_2_ heterostructures, attaining microsecond-scale response times (*τ* < 5 µs) with wavelength- and power-dependent photoresponse dynamics. Notably, Hu *et al.*^30^ realized a self-powered MoTe_2_/MoSe_2_ photodetector, achieving 0.72 A W^−1^ responsivity, 41.3% external quantum efficiency (EQE), and 7 × 10^11^ Jones detectivity at 638 nm, coupled with a rapid 120 µs response. This breakthrough provides critical material support for developing all-2D CMOS integrated circuits, aligning with emerging demands for low-power logic architectures and high-efficiency photo-electric conversion systems.

Despite these advances, key challenges remain for 2D MoTe_2_ photodetectors. First, achieving an ultralow dark current while maintaining high responsivity and efficiency, particularly in the visible to near-infrared (Vis-NIR) spectrum, is difficult. Second, extending the detection range to cover both Vis and NIR wavelengths without compromising overall performance is nontrivial.

In this work, we address these challenges by optimizing MoTe_2_-based photodetectors to achieve exceptionally low dark current and broadband detection capability from visible to near-infrared wavelengths. Through air annealing, we significantly improve the transport properties and reduce contact resistance, resulting in highly sensitive photodetectors. Notably, our devices exhibit a record responsivity at 808 nm and an EQE of over 600%, while maintaining ultralow dark current below 10^−13^ A at *V*_gs_ = −30 V. Additionally, the MoTe_2_/MoSe_2_ heterojunctions demonstrate excellent broadband photodetection, with peak responsivity at 671 nm and extended detection to 1064 nm, achieving high specific detectivity.

## 2. Experimental

### 2.1 Device fabrication

MoTe_2_ and MoSe_2_ flakes were mechanically exfoliated from their bulk crystals using tape (NITTO, 224S) and polydimethylsiloxane (Gel-Pak, PF). Subsequently, a conventional dry transfer technique was employed to construct the photodetector on a heavily p-doped Si substrate, where the stage was heated to 120 °C and maintained for 3 minutes to release the sample. Two Cr (5 nm)/Au (25 nm) electrodes, connecting to MoTe_2_ (source) and MoSe_2_ (drain), were fabricated using a laser direct writer (HWN-LDA-L4), with the Si/SiO_2_ substrate serving as a back gate. The channel length of the field-effect transistor is 2.5 µm. Finally, the device was annealed at 210 °C for 15 minutes in ambient air.

### 2.2 MoTe_2_/MoSe_2_ heterojunction device characterization

**Fig. 1:**
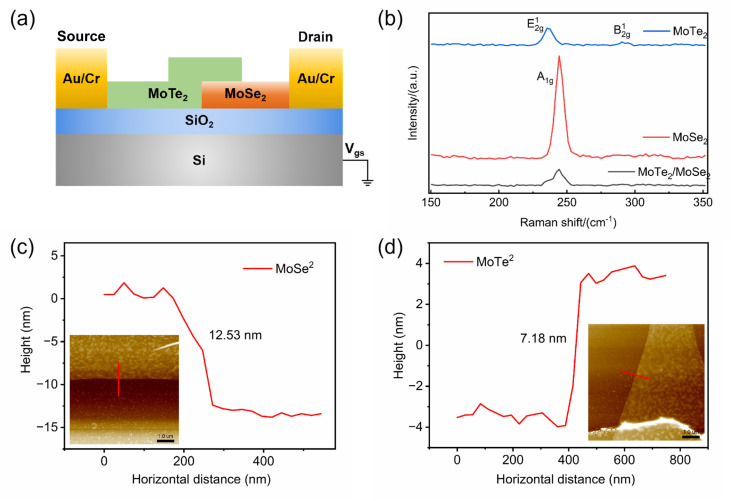
Structure and characteristics of MoTe_2_/MoSe_2_ heterojunction. (a) Structure of MoTe_2_/MoSe_2_ heterojunction device. (b) Raman mapping of MoTe_2_/MoSe_2_ heterojunction. (c) The thickness of MoSe_2_.Inset:the AFM image of MoSe_2_. (d) The thickness of MoTe_2_. Inset: the AFM image of MoTe_2_.

The material quality and thickness were characterized using a combination of Raman spectroscopy (Renishaw InVia plus with a 532 nm laser) and atomic force microscopy (AFM, Bruker Multimode 8HR). The Raman spectrum (Fig. 1b) confirms the crystal phase, showing characteristic peaks for 2H-MoTe_2_ (A_1g_ at 171 cm^−1^ and E_2g_ at 232 cm^−1^) and MoSe_2_ (A_1g_ at 242 cm^−1^). AFM measurements (Fig. 1c and d) reveal film thicknesses of 12.53 nm for MoSe_2_ and 7.18 nm for MoTe_2_. Based on the monolayer thickness (∼0.8 nm for MoSe_2_ and 0.7–0.8 nm for MoTe_2_ (ref. 31 and 32)), these correspond to approximately 15 and 10 layers, respectively, demonstrating precise layer control. For electrical testing, all measurements were conducted on a manual probe station (Lakeshore TTP4) equipped with a vacuum pump, a temperature control system, and a semiconductor characterization system (Tektronix Keithley 4200A-SCS), and all data were obtained at room temperature. Regarding the definitions of response times, the rise time typically refers to the time required for the detector's output current (or voltage) to increase from 10% to 90% of the steady-state dark current upon the onset of the optical signal; correspondingly, the fall time refers to the time period during which the output signal decreases from 90% to 10% of the steady-state photocurrent after the optical signal is turned off.

### 2.3 Noise analysis and detectivity calculation

The key figures of merit, including photoresponsivity (*R*_λ_), noise equivalent power (NEP), specific detectivity (D*), and external quantum efficiency (EQE), are calculated using the following formulas:
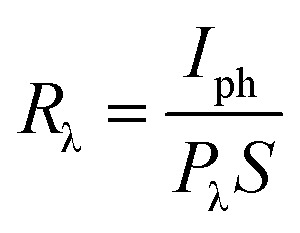

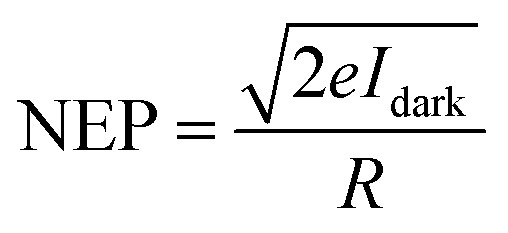

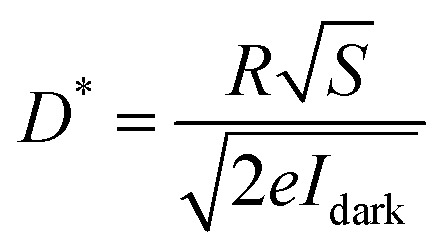

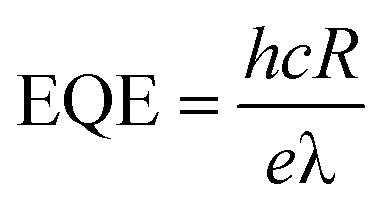
where *P*_λ_ represents the incident light power density, *S* and *e* are the effective area of the lateral homojunction and the unit electric charge, *I*_ph_ is the current value measured under illumination and *I*_dark_ is the dark current, *h* is the Planck constant, and *c* is the speed of light.

## 3. Results and discussion

### 3.1 MoTe_2_ FET

**Fig. 2:**
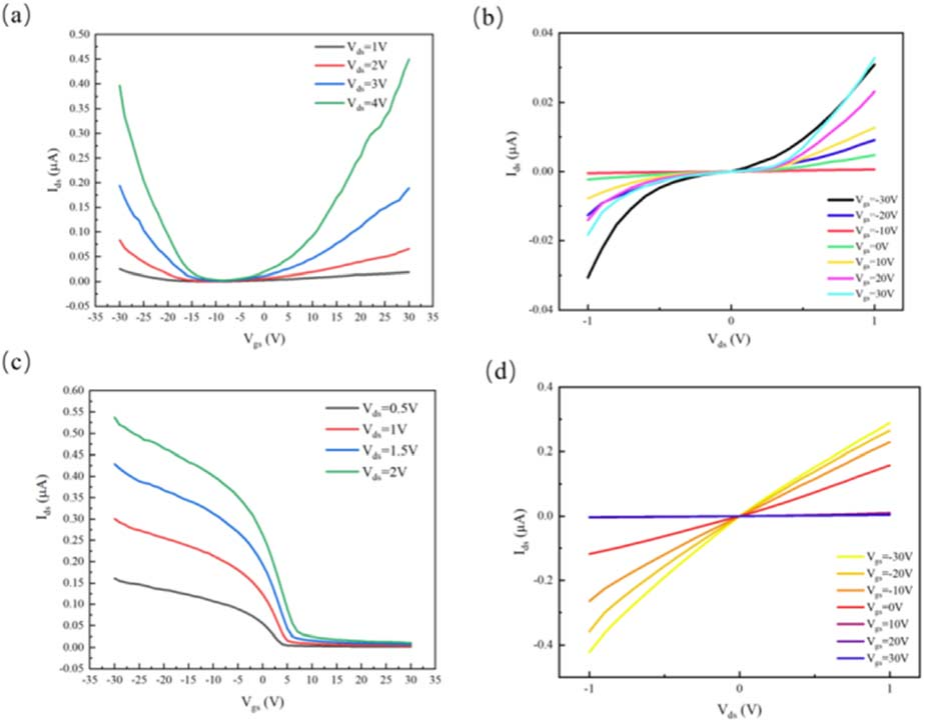
Electrical properties of MoTe_2_. (a) Pre-annealing transfer curve. (b) Pre-annealing output curve. (c) Post-annealing transfer curve. (d) Post-annealing output curve.

Electrical characterization of thin 2D MoTe_2_ devices revealed bipolar transport behavior, with dual conduction thresholds observed in the transfer curves under gate voltage sweeps (from −30 V to 30 V) (Fig. 2a). Enhanced nonlinearity in the output curves further confirmed the presence of Schottky contacts at the Cr/MoTe_2_ interfaces (Fig. 2b). A 15 minutes air annealing treatment at 210 °C induced transformative changes. Specifically, the Cr/MoTe_2_ contact transitioned from Schottky to ohmic behavior, with an 85% reduction in contact resistance, while the carrier polarity switched reversibly from n-type to p-type. This was accompanied by a 3.2× increase in hole mobility (from 14.3 cm^2^ V^−1^ s^−1^ to 46 cm^2^ V^−1^ s^−1^) (Fig. 2c and d).

This phenomenon is attributed to oxygen-mediated Te vacancy passivation, which reduced the free electron density (from 10^17^ to 10^15^ cm^−3^) and modulated Schottky barriers: the electron barrier increased by 0.14 eV, while the hole barrier decreased by 0.12 eV. Notably, thick MoTe_2_ films (35.8 nm), which initially exhibited n-type Schottky-limited transport, also achieved p-type conversion post-annealing, despite a weakened quantum confinement effect. This demonstrates the universality of the oxygen-intercalation mechanism in controlling the carrier polarity of MoTe_2_.

Stable p-type operation persisted across a wide range of bias voltages (−30 V ≤ *V*_gs_ ≤ 30 V, 1 V ≤ *V*_ds_ ≤ 3 V), confirming that air annealing is a robust and effective strategy for contact engineering and polarity control in 2D semiconductors.^33–35^ This approach addresses the Fermi-level pinning challenges in transition metal dichalcogenide (TMD) electronics, offering a practical route for enhancing device performance and enabling more versatile applications in optoelectronic and electronic systems.

### 3.2 MoTe_2_ photodetector

Photoresponse characterization across the 671–1064 nm spectral range demonstrated wavelength-optimized performance in MoTe_2_ photodetectors. The p-type transport was significantly enhanced under a −30 V gate bias as the light intensity increased.

**Fig. 3:**
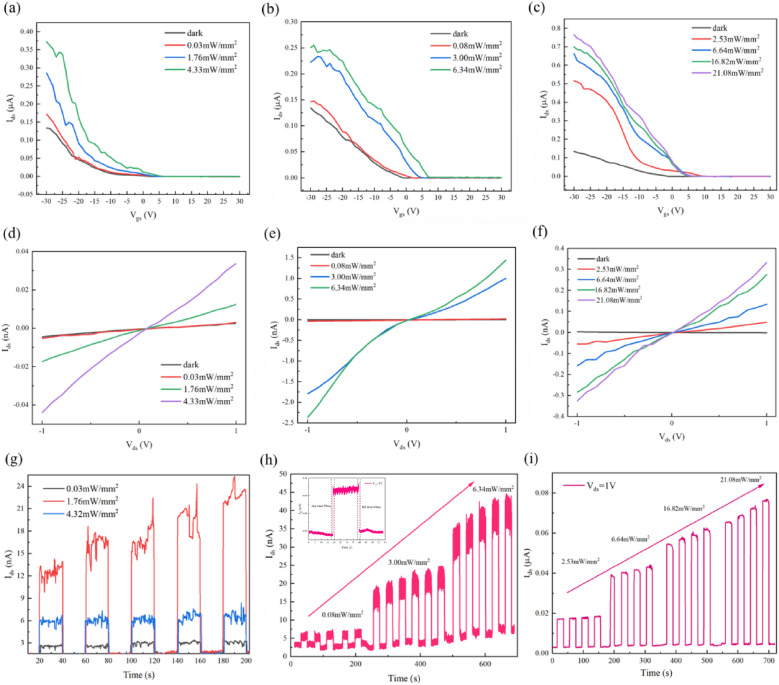
Optoelectronic properties of MoTe_2_ photodetector. (a–c) transfer curve under laser wavelength of 671 nm, 808 nm, 1064 nm respectively. (d–f) Output curve under laser wavelength of 671 nm, 808 nm, 1064 nm respectively. (g–i) *I*–*T* curve under laser wavelength of 671 nm, 808 nm, 1064 nm respectively.The inset shows the measured rise and fall times for the pure MoTe_2_ device under 808 nm laser irradiation.

**Fig. 4:**
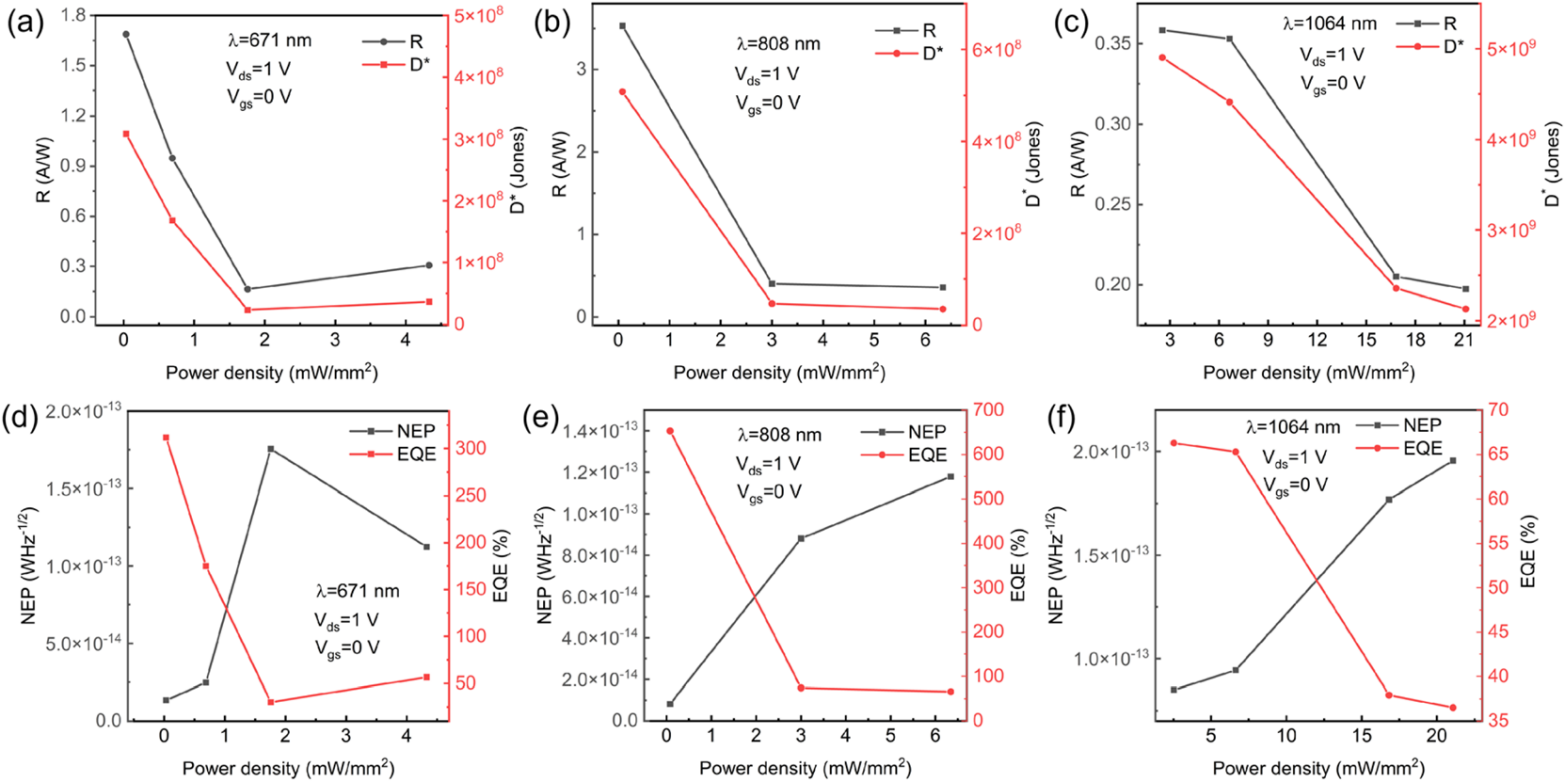
MoTe_2_ photodetector. (a–c) R and D under 671 nm, 808 nm, 1064 nm laser respectively. (d–f) NEP and EQE under 671 nm, 808 nm, 1064 nm laser respectively.

At 671 nm, the device exhibited well-balanced optoelectronic characteristics, achieving peak performance at 808 nm with a record responsivity of 3.53 A W^−1^, ultrawide noise equivalent power (NEP) of 8.20 × 10^−15^ W Hz^−1/2^, and exceptional EQE of 652%. The rise/fall times of the pure MoTe_2_ device is approximately 58 ms and 60 ms under 808 nm laser irradiation, respectively. Extension of operation to 1064 nm revealed a strong weak-signal detection capability, evidenced by a maximum photocurrent-to-dark-current ratio of 17.67 and a specific detectivity of 4.90 × 10^9^ Jones in Fig. 3 and 4.

Notably, an inverse correlation was observed between illumination intensity and key metrics: responsivity decreased by 68%, and detectivity dropped by 74% across the 0.01–1 mW mm^−2^ range. This behavior is attributed to trap-state saturation dynamics in the 2D system. Under low-intensity conditions, trap-mediated carrier lifetime enhancement (*τ*↑) led to photoconductive gain (*G*↑), resulting in increased responsivity (*R* ∝ *G*). However, at high intensities, the saturation of trap states reduced charge separation efficiency and increased recombination losses, causing a decrease in both responsivity and detectivity. This intensity-dependent quantum efficiency degradation,^36–38^ coupled with emerging thermal noise, establishes fundamental limits for gain optimization in low-dimensional photodetectors. These findings provide critical insights into trap-state engineering for advanced optoelectronic systems, informing future strategies for improving device performance. Besides, MoTe_2_-based photodetectors demonstrate considerable long-term stability and cyclic durability, a critical feature for practical applications.^39,40^

### 3.3 MoTe_2_/MoSe_2_ heterojunction photodetector

**Fig. 5:**
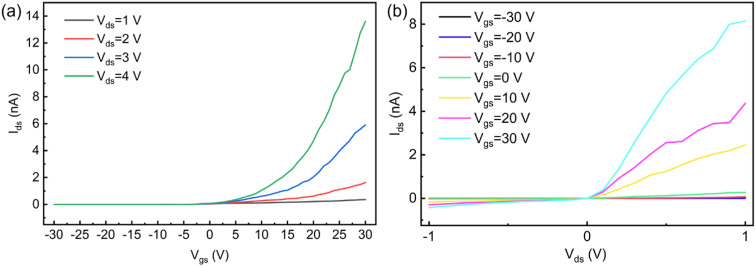
Electrical properties of MoTe_2_/MoSe_2_ heterojunction. (a) Transfer curve. (b) Output curve.

Electrical characterization of the MoTe_2_/MoSe_2_ heterojunction revealed n-type conductivity in the dark-state transfer curves (Fig. 5), with pronounced rectification behavior (rectification ratio = 23 at *V*_ds_ = ±1 V, *V*_gs_ = 0 V). This rectification arises from the type-II band alignment: the conduction band minimum (CBM) of MoTe_2_ (∼3.8 eV) and MoSe_2_ (∼4.1 eV) forms an interfacial built-in electric field, driving electron transfer from MoSe_2_ to MoTe_2_ and hole migration in the reverse direction under equilibrium conditions.

This charge redistribution results in upward band bending at the MoSe_2_ interface (electron depletion) and downward band bending at MoTe_2_ (hole depletion),^41^ establishing a voltage-dependent barrier modulation mechanism—forward bias reduces the interlayer barrier for electron transport, while reverse bias enhances it.

**Fig. 6:**
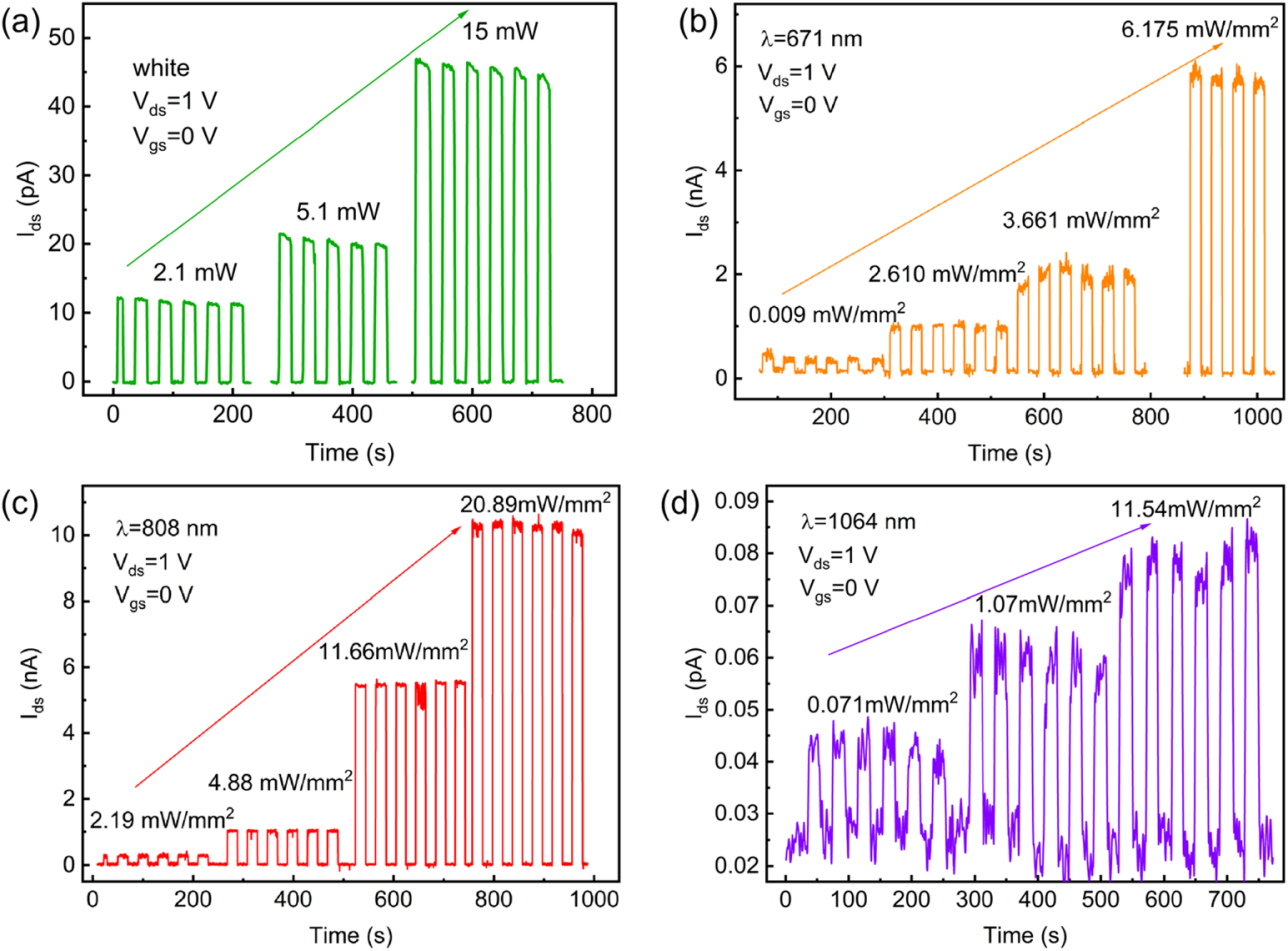
*I*–*T* curve of MoTe_2_/MoSe_2_ heterojunction photodetector. (a) White. (b) 671 nm. (c) 808 nm. (d) 1064 nm.

**Fig. 7:**
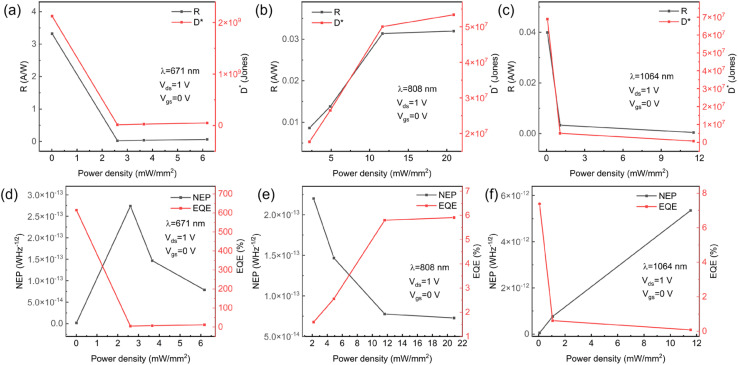
MoTe_2_/MoSe_2_ heterojunction photodetector. (a–c) *R* and *D* under 671 nm, 808 nm, 1064 nm laser respectively. (d–f) NEP and EQE under 671 nm, 808 nm, 1064 nm laser respectively.

The photoresponse evaluation across the visible to near-infrared wavelength range, as shown in Fig. 6 and 7, demonstrates broadband detection capabilities, with optimal photodetector performance at a wavelength of 671 nm. At this wavelength, the responsivity reaches 3.32 A W^−1^, with a quantum efficiency of 614% and an ultralow noise equivalent power (NEP) of 1.83 × 10^−15^ W Hz^−1/2^ under an optical power density of 0.009 mW mm^−2^.

**Fig. 8:**
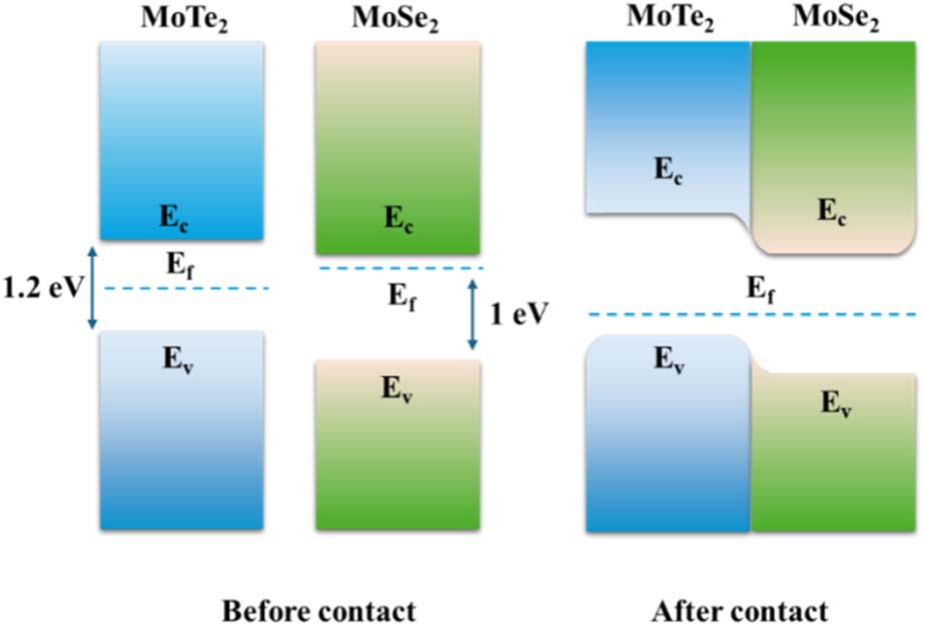
MoTe_2_/MoSe_2_ heterojunction band diagram.

The type-II heterostructure enhances carrier separation *via* the built-in electric field,^30,42,43^ achieving well-balanced rise/fall times of approximately 31 ms in Fig. 8. Furthermore, extended operation to 808 nm and 1064 nm confirms the spectral versatility of the device. The pure MoTe_2_ photodetector exhibits slower response (rise/fall times ≈ 58/60 ms) compared to the MoTe_2_/MoSe_2_ heterojunction device. This can be attributed to three main factors. First, the relatively low carrier mobility of pure MoTe_2_ increases the transit time for photogenerated carriers. Second, higher contact resistance at the metal-MoTe_2_ interface impedes efficient charge extraction. Finally, the presence of trap states or defects acts as recombination centers, prolonging the carrier lifetime and slowing the current decay. In contrast, the built-in electric field in the heterojunction promotes rapid charge separation and collection, thereby enhancing the response speed. Photodetectors based on thin MoTe_2_ layers exhibit superior performance metrics—such as higher responsivity, specific detectivity, and faster response times—compared to their thick-layer counterparts. This improvement primarily results from the higher surface-to-volume ratio, better electric field penetration, and enhanced charge transport in thinner layers, which collectively enable more efficient extraction of photogenerated carriers.^44^

However, responsivity and specific detectivity exhibited inverse correlations with light intensity: for instance, *R* decreased by 68% as light intensity increased from 0.01 to 1 mW mm^−2^. This is attributed to trap-state saturation: at higher intensities, the increased electron–hole pair density overwhelms the limited trap states, elevating recombination rates and reducing the effective carrier lifetime (*τ*↓). This leads to a decrease in photoconductive gain (*G*↓) and responsivity (*R* ∝ *G*), while additional shot noise further degrades D*. As summarized in Table 1, our MoTe_2_-based heterojunction photodetector demonstrates a superior overall performance profile—encompassing responsivity, EQE, specific detectivity, response time, and dark current—compared to other reported MoTe_2_-based devices.^28,30,44–46^

**Table 1:** Performance comparison of state-of-the-art MoTe_2_-based photodetectors

Materials	Light (nm)	*R*(A/W)	D* (Jones)	EQE	Response time	Ref.
MoTe_2_/SnSe_2_	365	2.05	4.04 × 10^9^	N/A	1.3/4.9 ms	45
SnSe_2_/Bi_2_Se_3_/MoTe_2_	808	0.493	1.8 × 10^11^	76%	553/583 µs	46
MoTe_2_	520	1.2	4.32 × 10^8^	285%	0.2/0.3 s	44
MoTe_2_/MoSe_2_	638	0.72	7 × 10^11^	41%	120/210 µs	30
MoTe_2_/MoSe_2_	White	1.5	2.7 × 10^12^	N/A	<30/<35 ms	28
MoTe_2_/MoSe_2_	671	3.32	2.12 × 10^9^	614%	31.2/31.3 ms	This work

## 4. Conclusions

In this study, we have demonstrated the substantial enhancement of 2D MoTe_2_ FETs through air annealing, which significantly improves electrical performance and stability. Air annealing at 210 °C for 15 minutes induced stable p-type conversion, leading to a 7.6-fold increase in transconductance (from 1.84 × 10^−9^ A V^−1^ to 1.40 × 10^−8^ A V^−1^), a 7.6-fold improvement in hole mobility (reaching 4.25 cm^2^ V^−1^ s^−1^), and a 5.8-fold boost in carrier density (to 7.83 × 10^11^ cm^−2^). These changes were accompanied by a transition from Schottky to ohmic contact, highlighting the significance of defect passivation in enhancing device performance.The optimized MoTe_2_ photodetector exhibited a peak responsivity of 3.53 A W^−1^ and 652% EQE at 808 nm, with ultralow noise characteristics (8.20 × 10^−15^ W Hz^−1/2^) under 0.08 mW mm^−2^ illumination, showcasing its potential for high-sensitivity photodetection.

Moreover, the MoTe_2_/MoSe_2_ type-II heterojunction demonstrated broadband detection capabilities, achieving a high responsivity of 3.32 A W^−1^, an EQE of 614%, and a response time of 31.2 ms at 671 nm. The heterojunction exhibited ultralow dark current (<10^−13^ A) and a rectification ratio of 23, indicating superior electrical characteristics. The 0.3 eV conduction band offset between MoTe_2_ and MoSe_2_ generated an efficient built-in electric field, facilitating effective carrier separation and yielding a 466 photocurrent-to-dark-current ratio under 15 mW white light. Notably, the inverse intensity dependence of responsivity (a 68% decrease from 0.01 to 1 mW mm^−2^) and detectivity were governed by trap-state-limited photoconductive gain, providing crucial insights into the performance limitations and fundamental design constraints of low-dimensional optoelectronic devices.

This work establishes air-annealed 2D MoTe_2_ as a tunable platform for visible-NIR photodetection and provides valuable guidelines for heterojunction interface engineering, paving the way for the development of next-generation optoelectronic systems with enhanced performance. Further studies on the long-term stability, endurance, and data retention of these devices will be essential to meet the lifetime and reliability requirements for practical applications.

## Author contributions

X. W.: conceptualization, investigation (fabrication of devices, Raman spectroscopy, IV measurements), visualization (device diagram drafting), data curation, writing – original draft preparation. Q. Y. Z.: visualization (device diagram drafting), data curation. J. K. S.: conceptualization, investigation (fabrication of devices, A. F. M., Raman spectroscopy, IV measurements), data curation, writing – original draft preparation. L. L.: writing – review & editing. H. L. T.: methodology, supervision. G. H. Z.: methodology, supervision. All authors: validation.

## Conflicts of interest

All authors declare no conflicts of interest.

## Data Availability

Most of the data presented in this paper is included in the main manuscript, and additional data are available from the corresponding author upon reasonable request.
